# Awareness of Nutrition Facts Labeling and Its Relation to Oral Health Practices and Caries Experience Among Adult Patients

**DOI:** 10.7759/cureus.50457

**Published:** 2023-12-13

**Authors:** Akram Qutob, Narmein Almashharawi, Shaza Hefni, Hassan Alzain, Mohamed Bamashmous, Dania Sabbahi

**Affiliations:** 1 Department of Dental Public Health, Faculty of Dentistry, King Abdulaziz University, Jeddah, SAU; 2 General Dentistry, Private Practice, Jeddah, SAU; 3 General Dentistry, King Abdulaziz Medical City, Jeddah, SAU; 4 Environmental Protection, Saudi Aramco Environmental Protection Organization, Dhahran, SAU

**Keywords:** saudi arabia, dietary practices, dental caries, oral health, nutrition facts label

## Abstract

Aim: To investigate the relationship between the usage of nutrition facts labels (NFL) and oral and dietary practices and the decayed, missing, and filled teeth (DMFT) score.

Methods: A self-administered questionnaire was distributed to a convenient sample of 150 adult dental patients attending the King Abdulaziz University Dental Hospital in Jeddah, Saudi Arabia. Electronic dental records were accessed to record each patient’s DMFT score.

Results: Only 38% (N=57) of the participants read the NFL on their food. A statistically significant association was found between patients' DMFT scores and the NFL reading. Participants who said they read NFLs had lower mean DMFT scores than those who said they did not (8.4 vs. 10.5).

Conclusions: The results of this study demonstrate that there is low usage of NFL among participants. A significant association was noted between the reading of the NFL and caries experience as measured using the DMFT index.

## Introduction

An estimated 90% of the world’s population is affected by some form of oral disease [1]. Dental caries and periodontal disease are the most common chronic oral diseases, affecting 3.9 billion people worldwide; the former accounts for 35% of all oral problems in adults, while the latter accounts for 10% [2,3]. It is, therefore, imperative that all sorts of preventive measures and good oral hygiene protocols are followed to avert the occurrence of these diseases in individuals.

Dental caries is considered a multifactorial disease with many risk factors: environmental, biological, and socio-behavioral [4]. One common culprit that has been implicated in many chronic systemic and oral diseases, including dental caries, is an unhealthy diet [5]. Dietary factors, especially the consumption of complex carbohydrates, have been conclusively proven to play a pivotal role in the development and progression of dental caries and, to a lesser extent, in periodontal diseases [6,7]. The frequency, quantity, and types of carbohydrates consumed have a significant effect on the level of progression of caries [8]. Furthermore, studies have shown that snacking outside of regular mealtimes is also associated with an increased incidence of caries [9,10]. Another common dental disease that is related to diet and nutrition is erosion, i.e., the progressive loss of hard tissue of the tooth due to frequent contact with acids. These acids can be either dietary acids or intrinsic acids in the body (from reflux, etc.), but studies have shown that dental erosion is most commonly caused by dietary acids from excessive consumption of sodas, alcoholic beverages, energy drinks, juices, citrus fruits, herbal teas, vinegar and pickles [11].

On the other hand, some foods like cheese, whole grains, peanuts, and cow’s milk are proven to either be associated with a low incidence of caries or to have a caries-preventative nature [7]. Good nutrition and good dietary habits also promote the development of healthy teeth and gingiva, as both macronutrients and micronutrients have an influence on enamel and dentin development and mineralization and on the overall development and protection of teeth [12].

Studies have been conducted on patients’ awareness of the effect of diet on oral health. These studies serve the purpose of offering valuable insights into the level of patient awareness and helping to determine if more is needed to be done to educate patients about the effect of diet on oral health. A study conducted in Palestine among 120 adult patients assessed their level of awareness regarding nutrition and oral health. This study also assessed the relationships between the nutritional status of the participants, their oral health practices, and their oral health condition. The results of this study revealed poor awareness among the participants, with females exhibiting a higher level of nutrition awareness than males [12]. A similar study was also conducted on a larger sample of 683 adult patients in Riyadh, Saudi Arabia. This study assessed patients’ awareness about the effect of diet on dental caries. The results of this study indicated that more than half (59%) of the participants knew the relationship between diet and dental caries [13]. Recently, Sachdev et al. [14] conducted a study on the correlation between dental nutrition knowledge and socioecological factors in the development of dental caries in 220 women from low-income housing communities in Texas, USA. The Dental Nutrition Knowledge Competency (DNKC) scale, a validated instrument consisting of 24 items, was used as the method of knowledge assessment for that study. The Decayed, Missing, and Filled Teeth due to caries (DMFT) index was used to assess the caries experience. The results of the study revealed limited dental nutritional knowledge among participants. The results also indicated an association between dental nutritional knowledge and dental caries after adjusting for demographic variables (p = 0.017).

The nutrition facts label (NFL) is one of the effective tools for communicating nutritional information to consumers. The nutritional information (e.g., nutritional content, list of ingredients, serving size, and calories per serving) allows the consumers to make informed decisions in order to achieve a healthy diet [15]. The research in this area that focuses on reading and understanding the nutrient facts labels is limited, with only a few studies addressing the effect of reading the NFL on a consumer’s diet [16-19]. However, none of these studies assessed the knowledge and awareness of participants regarding the NFL and its effect on their oral health status. Thus, the current study aimed to assess NFL usage and dietary practices among patients attending King Abdulaziz University Dental Hospital. Moreover, the relationships between the usage of NFL and the participant’s demographics, oral health practices, dietary practices, and participant’s caries experience were assessed. In addition, the relationships between dietary practices and the participant’s caries experience were explored.

## Materials and methods

Subjects/sample size

The present research is a cross-sectional study conducted at the Faculty of Dentistry, King Abdulaziz University, Jeddah, Saudi Arabia. Ethical approval for the study was obtained from the Research Ethics Committee at the Faculty of Dentistry, King Abdulaziz University (approval number 023-02-22).

The participants in the study were recruited as a convenient sample from new adult patients aged 18-60 years who attended the King Abdulaziz University Dental Hospital for dental treatment. The sample size was calculated using an online calculator for multiple linear regression (https://www.danielsoper.com/statcalc/calculator.aspx?id=1). The calculation was aimed at detecting a medium effect size (Effect size (f2) = 0.5) for the DMFT score as a dependent variable with age, gender, income, and NFL usage as predictors at 80% power and 5% significance. The calculation yielded a sample size of 84. Patients who were on special diets, couldn’t read Arabic or English, or had mental disorders, congenital oral diseases, or debilitating health conditions were excluded.

Questionnaire

A questionnaire was developed by the authors. Some of the questions were based on previous surveys with some modifications [12,20]. The questionnaire was divided into two sections, which consisted of both closed-ended and open-ended questions. The first section asked the participants about their demographic information, including age, gender, nationality, and education level. The second section contained items pertaining to oral health practices and dental attendance patterns, as well as dietary-related knowledge and habits. The NFL usage was assessed by asking the participants if they read the NFL before purchasing food items. The questionnaire was developed in the Arabic language. An English version was available as well for non-Arabic speakers and was developed via a forward and backward translation method for clarity and content validity.

Oral health assessment

The participants were approached during the undergraduate clinical sessions. Informed verbal consent was obtained prior to giving out the survey instrument, as well as written consent forms signed by the participants. All the personal information of the participants was kept confidential. Patients’ electronic dental records were accessed to calculate their DMFT scores. The dental charts in these records were filled out by the senior undergraduate dental students who recorded caries on the standard dental unit using an explorer and mirror, based on the International Caries Detection and Assessment System (ICDAS) scoring system. The teeth were first cleaned and dried using the 3-in-1 syringe before examination. Bitewing radiographs were used to assess interproximal caries. All dental charting was completed within eight weeks of completing the questionnaire. A tooth was recorded as decayed if a score of 2 or higher was recorded for any of the surfaces. A tooth was recorded as missing only if it was lost due to caries. Teeth missing due to periodontal disease or trauma were not included in the DMFT calculation. The treating student was approached by the research team to determine the causes of the tooth loss.

Data analysis

Data were entered into a software program (Microsoft Office Excel, Redmond, WA) and then imported into the Statistical Package for Social Sciences (version 25; IBM Corp., Armonk, NY). Descriptive statistics were used for calculating the frequency distributions of the categorical variables and the means and standard deviations for DMFT. Data normality was assessed using the Kolmogorov-Smirnov test. The data were found to be normally distributed. A Chi-square or Fisher’s exact test was used to assess the associations between the reading of NFL and different demographics, oral health practices, dental attendance patterns, and dietary-related variables. Independent t-tests and one-way analysis of variance (ANOVA) tests were used to compare the mean DMFT among the categories of dietary-related variables. Multiple linear regression analysis was used to control for the effect of demographic and socioeconomic variables on the relationship between NFL usage and DMFT score. Gender, income, and educational level were entered as categorical variables. All statistical analyses were two-tailed at a significance level of 0.05.

## Results

Sample characteristics

A hundred and fifty participants agreed to participate in the study. About 68% (N=102) of the participants were female, and 44.7% (N=67) of them were between the ages of 18 and 25 years. About half of the participants were single (54.7%, N=82), and 69.3% (N=104) had a university degree. The majority of the participants (79.3%, N=119) were Saudi, and 51.3% (N=77) reported a monthly income of less than 5000 Saudi Riyals. Table 1 summarizes the demographic characteristics of the sample.

**Table 1:** Relationships between reading nutrition facts labels (NFL) and demographic variables *The data have been represented as N and % **Using Chi-square or Fisher’s exact test; significant comparisons are in bold, the value at which the p-value is considered significant is (p<0.05). SAR=Saudi Riyals

Variables		Do you read nutrition facts labels?					p-value**
		Total*	Yes		No		
		N (%)	N	%	N	%	
Age	18-25	67 (44.7)	28	41.8	39	58.2	0.038
	26-35	42 (28.0)	20	47.6	22	52.4	
	36 or more	41 (27.3)	9	22.0	32	78.0	
Gender	Female	102 (68.0)	39	38.2	63	61.8	0.931
	Male	48 (32.0)	18	37.5	30	62.5	
Marital status	Single	82 (54.7)	33	40.2	49	59.8	0.678
	Married	64 (42.7)	22	34.4	42	65.6	
	Divorced or widowed	4 (2.7)	2	50.0	2	50.0	
Education	High school or less	46 (30.7)	13	28.3	33	71.7	0.102
	University or higher	104 (69.3)	44	42.3	60	57.7	
Income (SAR)	0 -4999	77 (51.3)	29	37.7	48	62.3	0.983
	5000 – 9999	33 (22.0)	13	39.4	20	60.6	
	10000 or more	40 (26.7)	15	37.5	25	62.5	
Nationality	Saudi	119 (79.3)	45	37.8	74	62.2	0.927
	Non-Saudi	31 (20.7)	12	38.7	19	61.3	

About 57% (N=86) of participants brushed their teeth at least twice a day. The majority of the participants (53.3%, N=80) brushed their teeth in the morning and before going to bed, most of them (76%, N=114) using a regular toothbrush as compared to an electric toothbrush (12.7%, N=19) while very few (2.7%, N=4) used a combination of both. Most participants (53.3%, N=80) brushed their teeth for 2-3 minutes, and most (78%, N=117) used fluoridated toothpaste. Many participants (44%, N=66) only visited the dentist when they experienced pain, while very few of them (5.3%, N=8) had never visited a dentist at all. Approximately 43% (N=64) of participants used dental floss once daily, while 38% (N=57) never used it. However, most participants (62%, N=93) used mouthwash once or twice daily. Table 2 summarizes the oral hygiene practices among the participants.

**Table 2:** Relationships between reading nutrition facts labels (NFL) and dental hygiene habits *The data have been represented as N and % **Using Chi-square or Fisher’s exact test; significant comparisons are in bold, the value at which the p-value is considered significant is (p<0.05).

Variables		Do you read nutrition facts labels?					p-value**
		Total*	Yes		No		
		N (%)	N	%	N	%	
Brushing Frequency	Never	2 (1.3)	1	50.0	1	50.0	0.478
	Once a day	35 (23.3)	11	31.4	24	68.6	
	Twice a day	86 (57.3)	37	43.0	49	57.0	
	Three times or more a day	27 (18.0)	8	29.6	19	70.4	
Brushing Duration	Less than two minutes	66 (44.0)	21	31.8	45	68.2	0.075
	2 to 3 minutes	80 (53.3)	36	45.0	44	55.0	
	More than 5 minutes	4 (2.7)	0	0.0	4	100.0	
Frequency of Dental Visits	Never	8 (5.3)	2	25.0	6	75.0	0.074
	Every 3 months	11 (7.3)	5	45.5	6	54.5	
	Every 6 months	25 (16.7)	7	28.0	18	72.0	
	Every 12 months	30 (20.0)	17	56.7	13	43.3	
	More than 1 year	10 (6.7)	6	60.0	4	40.0	
	On pain only	66 (44.0)	20	30.3	46	69.7	
Flossing Frequency	Never	57 (38.0)	21	36.8	36	63.2	0.322
	Once a day	64 (42.7)	28	43.8	36	56.2	
	Two times or more daily	29 (19.3)	8	27.6	21	72.4	
Mouthwash Using Frequency	Never	51 (34.0)	14	27.5	37	72.5	0.061
	Once or twice a day	93 (62)	42	45.2	51	54.8	
	More than twice a day	6 (4.0)	1	16.7	5	83.3	

The majority of participants ate fruits (91.3%, N=137) and vegetables (96.7% (N=145); 76% (N=114) said yes, while 20.7% (N=31) said sometimes). Many participants ate fruits (63.3%, N=95) and vegetables (44%, N=66) only some days a week, while only a few ate fruits (21.3%, N=32) and vegetables (35.3%, N=53) once daily, and fewer ate fruits (10%, N=15) and vegetables (17.3%, N=26) twice or more daily.

A vast majority of the participants (81.3%, N=122) had never eaten brown rice. However, 46%, (N=69) of the participants reported always eating brown bread. The majority of the participants reported that they always drink milk and eat milk products, meat, fish, and nuts (81.3% (N=122), 76% (N=114), 66% (N=99), and 55.6% (N=83), respectively). More than half of the participants (51.4%, N=77) reported that they did not know or were unsure about the difference between saturated and unsaturated fats. Many participants (36%, N=54) have four to six glasses of water daily, while very few participants (9.3%, N=14) drink less than one glass of water daily.

In the present study, 58% (N=87) of the participants reported that they always eat some of their meals from fast food restaurants, and about 74% (N=112) reported that they always cook some of their meals at home. The majority of the participants (62%, N=93) reported that they do not monitor their caloric intake. Similarly, about 73% (N=110) of the participants stated that they did not know or were unsure how to calculate food portions. Only 34.9% (N=52) of participants considered most of their food to be healthy, and only 38% (N=57) read the NFL on their food. Table 3 depicts the dietary habits of participants.

**Table 3:** Relationships between DMFT and dietary-related variables DMFT= decayed, missing, and filled teeth *The data have been represented as N and %. **Using independent t-test and one-way ANOVA; significant comparisons are in bold, the value at which the p-value is considered significant is (p<0.05).

Dietary habits*			DMFT			p-value**
Variables	N	%	mean	SD	Median	
Do you eat fruits?						
No	13	8.7	10.6	5.0	10.0	0.537
Yes	137	91.3	9.6	5.4	10.0	
Do you eat vegetables?						
No	5	3.3	11.4	8.0	10.0	0.739
Sometimes	31	20.7	9.83	6.5	10.0	
Always	114	76	9.54	5.0	10.0	
Do you eat brown bread (flat)?						
No	44	29.3	10.1	4.9	10.5	0.394
Sometimes	37	24.7	8.6	6.3	8.0	
Always	69	46.0	10.0	5.1	10.0	
Do you eat brown rice?						
No	122	81.3	10.0	5.5	10.0	0.067
Sometimes	18	12.0	7.0	4.9	7.5	
Always	10	6.7	11.0	3.4	11.0	
Do you eat milk and milk products?						
No	12	8	10.5	4.9	11.5	0.200
Sometimes	16	10.7	11.8	5.2	12.5	
Always	122	81.3	9.3	5.4	9.0	
Do you eat meat?						
No	10	6.7	13.0	4.9	13.5	0.020
Sometimes	26	17.3	11.2	6.7	10.5	
Always	114	76	9.0	4.9	9.0	
Do you eat fish?						
No	20	13.3	10.5	5.9	11.0	0.009
Sometimes	31	20.7	12.0	5.8	12.0	
Always	99	66	8.8	4.9	9.0	
Do you eat nuts?						
No	26	17.4	12.2	7.9	12.0	0.021
Sometimes	40	26.8	9.6	4.5	10.0	
Always	83	55.6	8.8	4.5	9.0	
Do you know the difference between saturated and unsaturated fats?						
No	52	34.7	11.3	4.9	12.0	0.016
Not sure	25	16.7	9.4	4.6	10.0	
Yes	73	48.6	8.6	5.7	8.0	
How many glasses of water do you drink a day?						
Less than 1 glass	14	9.3	12.6	5.9	13.5	0.040
2 - 3 glasses	51	34	10.4	5.4	10.0	
4-6 glasses	54	36	8.4	5.1	8.0	
More than 6 glasses	31	20.7	9.4	5.0	10.0	
Do you eat fast food?						
No	18	12	10.1	5.4	11.5	0.919
Sometimes	45	30	9.8	4.6	10.0	
Always	87	58	9.5	5.8	9.0	
Do you cook food at home?						
No	12	8.0	9.6	4.4	10.0	0.897
Sometimes	26	17.3	10.1	7.1	10.0	
Always	112	74.7	9.6	5.0	10.0	
Do you monitor calories when you eat?						
No	93	62.0	10.6	5.1	11.0	0.023
Sometimes	28	18.7	7.7	5.6	7.5	
Always	29	19.3	8.6	5.5	8.0	
Do you know how to calculate food portions?						
No	80	53.3	10.3	4.9	10.5	0.206
Not sure	30	20.0	9.6	4.9	9.0	
Yes	40	26.7	8.5	6.4	8.5	
Do you consider that most of your food is healthy?						
No	62	41.6	9.7	6.0	9.5	0.417
Not sure	35	23.5	10.4	5.2	11.0	
Yes	52	34.9	8.9	4.6	10.0	
Do you read nutrition facts labels?						
No	93	62	10.5	5.4	10.0	0.020
Yes	57	38	8.4	5.1	8.0	

The association between reading NFL and other variables

The associations between the reading of the NFL and demographics, oral health practices, and dietary habits are summarized in Tables 1, 2 and Table 4, respectively. Statistical analyses revealed a significant association between the age of the participants and the reading of the NFL. A higher percentage of participants younger than 36 years old reported that they read the NFL in comparison to the participants older than 36 years. No statistically significant association was noted between the other demographic variables and the reading of the NFL. Similarly, statistical analysis showed no statistically significant association between any of the variables related to oral hygiene practices and reading of the NFL. Significant associations were noted between some of the dietary-related variables and the reading of the NFL, including eating brown bread, knowing the difference between saturated and unsaturated fats, cooking food at home, monitoring caloric intake, and knowing how to calculate food portions. Higher percentages of participants who reported these dietary habits also stated that they read the NFL.

**Table 4:** Relationships between reading nutrition facts labels (NFL) and dietary-related variables *The data have been represented as N and %. **Using Chi-square or Fisher’s exact test; significant comparisons are in bold, the value at which the p-value is considered significant is (p<0.05).

Dietary habits	Reading the nutrition facts labels*				p-value**
	Yes		No		
	N	%	N	%	
Do you eat fruits?					
No	3	25.0	9	75.0	0.324
Yes	54	39.4	83	60.6	
Do you eat vegetables?					
No	2	3.5	3	3.2	0.761
Sometimes	10	17.5	21	22.6	
Always	45	78.9	69	74.2	
Do you eat brown bread (flat)					
No	10	22.7	34	77.3	0.017
Sometimes	13	35.1	24	64.9	
Always	34	49.3	35	50.7	
Do you eat brown rice?					
No	44	36.1	78	63.9	0.324
Sometimes	7	38.9	11	61.1	
Always	6	60.0	4	40.0	
Do you eat milk and milk products?					
No	4	33.3	8	66.7	0.844
Sometimes	7	43.8	9	56.3	
Always	46	37.7	76	62.3	
Do you eat meat?					
No	4	40.0	6	60.0	0.705
Sometimes	8	30.8	18	69.2	
Always	45	39.5	69	60.5	
Do you eat fish?					
No	9	45.0	11	55.0	0.133
Sometimes	7	22.6	24	77.4	
Always	41	41.4	58	58.6	
Do you eat nuts?					
No	10	38.5	16	61.5	0.060
Sometimes	9	22.5	31	77.5	
Always	37	44.6	46	55.4	
Do you know the difference between saturated and unsaturated fats?					
No	8	15.4	44	84.6	<0.001
Not sure	7	28.0	18	72.0	
Yes	42	57.5	31	42.5	
How many glasses of water do you drink a day?					
Less than 1 glass	5	35.7	9	64.3	0.744
2 - 3 glasses	20	39.2	31	60.8	
4-6 glasses	18	33.3	36	66.7	
More than 6 glasses	14	45.2	17	54.8	
Do you eat fast food?					
No	8	44.4	10	55.6	0.830
Sometimes	17	37.8	28	62.2	
Always	32	36.8	55	63.2	
Do you cook food at home?					
No	2	16.7	10	83.3	0.042
Sometimes	6	23.1	20	76.9	
Always	49	43.8	63	56.3	
Do you monitor calories when you eat?					
No	17	18.3	76	81.7	<0.001
Sometimes	18	64.3	10	35.7	
Always	22	75.9	7	24.1	
Do you know how to calculate food portions?					
No	18	22.5	62	77.5	<0.001
Not sure	12	40.0	18	60.0	
Yes	27	67.5	13	32.5	
Do you consider that most of your food is healthy?					
No	18	29.0	44	71.0	0.111
Not sure	14	40.0	21	60.0	
Yes	25	48.1	27	51.9	

The association between DMFT and dietary-related variables

The mean DMFT of the participants in the study sample was 9.7±5.4. The relationships between the DMFT and dietary-related variables can be seen in Table 3. A lower mean DMFT score was noticed for participants who reported that they always eat meat, fish, or nuts. Additionally, a statistically significant association was detected between DMFT scores and knowledge about the differences between saturated and unsaturated fats, with lower mean DMFT scores among the participants who stated that they understood this difference. Moreover, significantly lower DMFT scores were noted in participants who drank four to six glasses of water in comparison to those who drank less than one glass of water, and among participants who monitored their calorie intake. A statistically significant association was found between reading NFL and DMFT, with lower mean DMFT scores for participants who reported that they read the NFL in comparison to those who did not (8.4 and 10.5, respectively). The association between NFL reading and DMFT scores remained significant after adjusting for the demographic and socioeconomic variables (Table 5).

**Table 5:** Multiple linear regression model for controlling the effect of demographic and socioeconomics variables on the relationship between NFL reading and DMFT scores NFL=nutrition facts labels; DMFT=decayed, missing and filled teeth. Significant associations are shown in bold.

Independent variables	B	SE	Beta	Significance
Gender (Female=0, Male=1)	-0.94	0.916	-0.008	0.243
Income (<10000 SAR=0, ≥10000 SAR=1)	-3.289	0.993	-0.272	0.001
Education level (High school or less=0, University or higher=1)	-0.293	0.950	-0.025	0.758
NFL reading (Yes=0, No=1)	2.076	0.871	0.188	0.018

## Discussion

To the best of the authors’ knowledge, this is the first study conducted in Saudi Arabia that assessed the relationship between patients’ usage of NFL and their oral hygiene practices, dietary habits, and caries experience. The caries experience of the participants was examined using the DMFT index, which has been in use globally for over 70 years as the most reliable indicator for assessing the oral health of patients, especially in epidemiological studies [21]. The DMFT index is simple to learn and use, and it has good validity, reliability, and reproducibility [22].

The mean DMFT score of participants in the present study was 9.7±5.4 (range 0-32), which indicates high caries experience and poor oral hygiene status for participants in the study. Previous population studies have assessed caries experience using DMFT in Saudi Arabia, and most of them reported a high prevalence and high caries risk with poor oral hygiene status [23]. A study conducted in Jeddah on 270 males aged 12-20 years found a mean DMFT of 2.1±2.77 [24]. Similarly, another study conducted by Al-Hebshi et al. in Jazan found a mean DMFT score of 1.98±2.10 [25]. Al-Shahrani et al. conducted another study in Dammam and found a mean DMFT of 5.61±3.01 [26]. The lower mean DMFT scores in these studies can be attributed to the differences in the age range of the participants in these studies compared to the current study. These studies were conducted on participants with permanent teeth below 20 years of age, while the age range of the participants in the current study ranged between 18 and 60 years. Another study with a similar age range reported slightly higher mean DMFT scores than the present study; the study, conducted by Al-Mobeeriek A on adults aged 20-50 years, demonstrated a mean DMFT of 13.81 in participants with psychological disorders and 10.48 in participants without psychological disorders [27].

The present study showed satisfactory brushing habits among participants, with most of them (75.3%, N=113) brushing their teeth twice or more daily. Tooth brushing frequency has been shown to have an effect on the initiation and development of carious lesions, as well as periodontitis [28]. Low brushing frequency is associated with a greater risk of caries and periodontal diseases, and studies indicate that brushing less than twice a day causes a statistically significant increase in incremental carious lesions compared to more frequent brushing (standardized mean difference (SMD): 0.34; 95% CI: 0.18 to 0.49 [29]. The use of dental floss and mouthwash was very low in the present study, which may be one of the reasons why DMFT was high and poor oral hygiene was noted among the participants in the current study.

The frequency and pattern of dental visits indicate the participants' oral hygiene. In the present study, the majority of participants were non-regular attendees (56%, N=84), most of whom visited a dental clinic only when there was pain (44%, N=66). This may be one of the reasons why the mean DMFT score was high. A study by Crocombe et al. showed that non-regular attendees to dental clinics have higher DMF scores [30]. However, it must be noted that the world has only recently emerged from the coronavirus pandemic, and dental visits during the last few years have declined significantly due to general public anxiety and fear of developing acute respiratory disease upon visiting clinics and hospitals [31]. This could be one of the reasons why our study showed non-regular attendees, as the survey did not allow participants to mention their pre-pandemic habits.

Dietary habits were evaluated in our study and participants reported satisfactory dietary habits, with many participants eating fruits and vegetables frequently and most of them eating meat sparingly. As discussed previously, dietary factors are important predictors of oral health [6,7]. A recent study evaluated the relationship between eating habits and oral health. The study reported a significant association between the consumption of vegetables and fruits and caries experience, while no significant associations were noticed between the consumption of meat and fish and caries experience [32]. These findings contrast with the present study, which found no association between the consumption of fruits and vegetables and caries experience but did find a significant association between the consumption of meat and fish and caries experience. The present study found no relationship between the consumption of dairy products and caries experience. This is in contrast with the study conducted by Moynihan PJ, which found that foods like dairy and dairy products (especially cow’s milk), whole grains, and peanuts are associated with either a low incidence of caries or a caries-preventive nature [7]. On the other hand, the results of the present study revealed a statistically significant association between the eating of nuts and DMFT scores, which confirms the findings of Moynihan PJ.

In the present study, less than half of the participants monitor their caloric intake (38%, N=57). A significant association was noted between the monitoring of this intake and a participant’s DMFT score, with participants who did not monitor their caloric intake having higher mean DMFT scores than those who did. These findings agree with a recent study which found that participants who consume fewer calories are less likely to develop caries [32].

One of the main objectives of this study was to evaluate the usage of NFL among the participants. We found that 38% (N=57) of the study sample regularly read NFLs. This is in accordance with another study that was conducted in Saudi Arabia, in which about 37% of the participants reported that they always or sometimes read the NFL [20]. Two studies in South Korea and the United States reported lower (22%) and higher (59%) usage of the NFL among the participants, respectively [17,19]. In the current study, reading the NFL was associated with a lower mean DMFT score. To the best of our knowledge, this study is the first to assess the effects of reading the NFL on oral hygiene status. Thus, we did not have findings from previous studies to compare our results with.

Labelling foods and providing NFL to consumers is considered an effective and important strategy to reduce the amount of dietary sugar intake, by converting nutritional information to informed consumer choices that are aimed at healthy food and beverage consumption [33]. Recently, Jeydjevic et al. conducted a study using random microsimulation estimation models to study the effects of food package labelling on caries in the German population. Their findings concluded that front-of-package food labelling has the potential to substantially reduce caries risk, caries progression, caries-related morbidity, and economic burden [34]. As expected, the results of the current study revealed a significant association between reading NFL and good dietary knowledge and habits. Participants who reported reading the NFL indicated that they tend to monitor their caloric intake, calculate their food portions, cook at home, and know the difference between saturated and unsaturated fats.

The major limitation of the present study was that all participants were recruited as a convenient sample from a single center. This can lead to selection bias and limit the generalizability of the results beyond this specific sample. Secondly, the cross-sectional nature of this study precludes the determination of causality between the observed variables. Future longitudinal studies are needed to establish a temporal sequence and causation. In addition, the use of self-reported questionnaires might result in the development of biases related to social desirability and recall biases. Furthermore, there was no screening for any systemic conditions; the eligibility criteria in the study excluded patients with debilitating health conditions, but there was no scope for taking a detailed history or screening early-stage systemic diseases like diabetes. So, some of the participants could have been pre-diabetic or diabetic and these could have been the outliers in the DMFT score. In addition, other confounding factors of poor oral hygiene, like cigarette smoking and other harmful habits, were not accounted for. Finally, the undergraduate students who performed the dental examination were not calibrated for this research specifically. This is not expected to affect the validity of the results as all students were trained to follow certain examination protocol and all charting were checked and verified by experienced instructors.

## Conclusions

The present study investigated the relationship between the usage of nutrition facts labels (NFL) and oral and dietary practices and the decayed, missing, and filled teeth (DMFT) score. In this specific study sample, 38% (N=57) of participants read nutritional fact labels (NFL), which was significantly associated with lower caries experience, as indicated by mean DMFT scores of 8.4 in NFL readers compared to 10.5 in non-readers. These findings suggest a need for increased community education to enhance NFL usage and nutritional knowledge.
